# Age-dependent differences in type I interferon, IL-12 and pro-inflammatory cytokine production by porcine peripheral blood mononuclear cells in response to pseudorabies virus-infected cells

**DOI:** 10.3389/fimmu.2025.1596490

**Published:** 2025-07-03

**Authors:** Manon S. C. Claeys, Simon G. A. L. Brabant, Stijn Rosschaert, Cliff Van Waesberghe, Herman W. Favoreel

**Affiliations:** Laboratory of Immunology, Department of Translational Physiology, Infectiology and Public Health, Faculty of Veterinary Medicine, Ghent University, Merelbeke, Belgium

**Keywords:** pseudorabies virus, innate immunity, neonates, peripheral blood mononuclear cells, plasmacytoid dendritic cells, interferon-α, Th1 immunity, immaturity

## Abstract

Pseudorabies virus is a porcine alphaherpesvirus that causes devastating disease with high mortality in young piglets but much milder, mainly respiratory, problems in older pigs. Here, we report marked age-dependent differences in the cytokine response profile of primary porcine peripheral blood mononuclear cells (PBMCs) to pseudorabies virus (PRV)-infected cells. Notably, IFN-α and IL-12 production in response to PRV remains low to almost undetectable in piglets up to 8.5 weeks of age, followed by a marked increase in older piglets, which coincides with age-related shifts in PRV symptomatology. Additionally, we found that PBMC from newborn piglets exhibit a high IL-6 and IL-10 production which, in combination with the low IL-12 levels, suggests a suppressed Th1 immune response, similar to neonatal humans and mice. Our results reveal a remarkable age-dependent difference in PBMC cytokine response to PRV and provide a basis to identify cytokines or adjuvants that shift the neonatal immune response towards a Th1 response, potentially improving outcomes of severe viral infections in the neonatal period in both pigs and humans.

## 1 Introduction

Suid alphaherpesvirus 1, also known as pseudorabies virus (PRV), is an alphaherpesvirus that has the pig as its natural host (1). PRV is highly infectious and the causative agent of Aujeszky’s disease, which leads to large economic losses in the swine industry worldwide. Thanks to intensive vaccination and eradication programs, PRV has been eradicated from the domesticated swine population in numerous countries, such as New Zealand, Mexico, Canada, the US and large parts of Europe (2, 3). Nevertheless, it is still endemic in different regions and new, highly virulent strains have emerged in China, posing a constant threat to the swine populations worldwide (4, 5). Symptomatology of PRV infection is highly age dependent. Young piglets (<9 weeks) suffer from severe neurological symptoms, with extremely high mortality (up to 100%) in piglets under 3 weeks of age. From 9 weeks onwards, clinical presentation shifts to a much milder respiratory disease (sneezing, nasal discharge, cough). In adult pigs, PRV infection is often subclinical, except in pregnant sows, where PRV infection may lead to reproductive problems (abortion, still birth) (6–9).

An age-dependent shift in severity of symptomatology can also be observed in the related human alphaherpesvirus, herpes simplex virus (HSV1&2). In neonates, HSV infections may cause systemic inflammation and an often fatal encephalitis, while in immunocompetent adults infection is frequently asymptomatic, or causing relatively mild symptoms like cold sores, also known as localized skin, eye and mouth disease (SEM) (10).

Why younger individuals appear to be much more susceptible to severe alphaherpesvirus infections remains to be elucidated.

On the one hand, many studies report that a robust type I interferon (IFN-I) production early in infection is crucial to suppress viral replication and severe pathology in herpesvirus infections (11–14). Plasmacytoid dendritic cells are specialized in sensing viral DNA and RNA and subsequently producing large amounts of the antiviral IFN-I (15). Even though pDC only represent a small percentage (0.1-0.5%) of the nucleated blood cells, they produce up to 1,000 times more IFN than any other cell type (15–18). Importantly, both *in-vitro* and *in-vivo*, pDCs are the major source of IFN-α production by peripheral blood mononuclear cells (PBMCs) in response to alphaherpesviruses (19, 20). Low pDC counts or deficiencies in IFN-α production are associated with increased susceptible to severe recurrent herpesvirus infections, further underlying the importance of a robust IFN-α response to control alphaherpesviruses (12, 13).

On the other hand, both for PRV and HSV, severe symptoms in young individuals are linked to a specific and lethal systemic host inflammatory response (10, 21). IL-6, TNF-α, and IL-1-β are critical pro-inflammatory cytokines and their production needs to be tightly regulated, as excessive and uncontrolled production can lead to an overwhelming systemic inflammatory response that leads to widespread tissue damage, organ dysfunction, and even death. Proper control of these pro-inflammatory cytokine levels is thus essential to ensure their protective roles without triggering detrimental systemic effects (6, 21–23).

In addition, another important cytokine in generating a long-lasting effective antiviral immune response is IL-12. Dendritic cells and monocytes are the main producers of IL-12. Apart from stimulating lytic activity by NK cells, IL-12 stimulates proliferation of IFNgamma-producing T cells and steers the immunity towards a Th1 biased response (24–26). Such Th1 response is crucial to effectively protect against herpesviruses, and adequate IL-12 production during infection or vaccination is essential in generating effective long lasting cellular immunity (27).

Currently, it is not known whether there are age-dependent differences in the ability of PBMC/pDC to produce any of these critical cytokines (IFN-α, IL-6, TNF-α, and IL-1-β and/or IL-12) in response to -α herpesviruses. Hence, the aim of the present study was to assess whether there are age-dependent differences in the production of IFN-α as well as other relevant immunomodulatory/immunostimulatory cytokines (IL-12, IL-6, IL-1-β, and TNF-α and IL-10) by porcine PBMC in response to PRV-infected cells, and whether this may correlate with the age-dependent differences in PRV-associated disease progression and severity. A longitudinal study was performed using PBMC from neonatal pigs (4 days of age) till well into puberty (28.5 weeks of age). We report that PBMC from young piglets display a very poor to undetectable IFN-α response to PRV (or a TLR9 agonist), whereas this response increases dramatically between 8.5 and 12.5 weeks of age, a time window that correlates with the marked reduction in PRV symptomatology. A similar trend was observed for IL-12, whereas levels of pro-inflammatory cytokines IL-6 and IL-1-β were increased in very young piglets (<= 4.5 weeks of age). Hence, this study reveals a marked age-dependent cytokine response in porcine PBMC in response to PRV or a TLR9 agonist, supporting the notion that age-dependent differences in cytokine responses may contribute to dramatic age-dependent differences in alphaherpesvirus disease.

## 2 Materials and methods

### 2.1 Cells and viruses

Swine testicle cells (ST) were cultured in Earle’s minimum essential medium (MEM) (Life Technologies) with 10% fetal calf serum (Serana), 1mM sodium pyruvate and antibiotics (100U/ml penicillin, 0.1 mg/ml streptomycin, 0.05mg/ml gentamycin) (Life Technologies).

Porcine PBMC were isolated from whole blood. Blood was collected from the right jugular vein from 4 day old to 28.5 week old piglets in 2 to 4 week increments up until 16.5 weeks of age and at 28.4 weeks of age. From 12 weeks onwards, the piglets were housed at the Faculty of Veterinary Medicine, Ghent University. PBMC were isolated using a lymphoprep density gradient (1.077g/L, Axis-Shield). Red blood cells were lysed using Tris-buffered ammonium chloride (NH_4_Cl 0.74% (Sigma) and Tris 0.2% (C_4_H_11_NO_3_ VWR)). Next, PBMC were washed and resuspended in ‘pDC medium’ (RPMI 1640 (Life Technologies) with 10% fetal calf serum (Serana), 1mM sodium pyruvate, 1mM non-essential amino acids (Serana), 20 μM β-mercaptoethanol (Life Technologies) and antibiotics (100U/ml penicillin, 0.1 mg/ml streptomycin, 0.05mg/ml gentamycin) (Life Technologies) (=pDC medium).

The PRV strains that were used in the current study were described before, Becker (28), Bartha (29). The wild type (WT) Becker strain was a kind gift of Prof. Dr. L. Enquist (Princeton University, USA). The attenuated Bartha vaccine strain was kindly donated by Prof. Dr. H. Nauwynck (Ghent University, Belgium).

### 2.2 PBMC incubation with infected cells

Confluent ST cells were inoculated with Bartha or Becker virus in a 96-well plate at a MOI of 10. At 2hpi, inoculum was washed away and 100μL of citrate buffer (40mM sodium citrate, 10mM KCl, 135 mM NaCl, [pH 3]) was added to the cell surface in each well for 2 min to inactivate non-entered virions. Next 500 000 PBMC were added in 200μL of pDC medium and co-incubated for 22h. To ensure consistency across experiments, PBMCs isolated from piglets at different ages were stimulated with the exact same virus stock, derived from a single production batch.

### 2.3 Reagents

CpG ODN32 was ordered from Integrated DNA Technologies (IDT) as described before (30).

### 2.4 ELISA

IFN-α concentrations were measured by ELISA. ELISA microplates (maxisorp, Thermo Scientific Nunc) were coated with a mouse anti-porcine IFN-α antibody F17 (5μg/ml in 0.1 M NaHCO3) overnight at room temperature. The plates were incubated for 1hr with blocking buffer (PBS with 0.05% Tween20 and 0.5% bovine serum albumin (Merck Millipore). Samples and standard (recombinant porcine IFN-α) were diluted in blocking buffer, added to the microplates and incubated for 2hr at room temperature. Then the microplates were washed, followed by incubation with the biotinylated mouse anti-porcine IFN-α K9 antibody for 1.5hr at room temperature. Then HRP conjugated streptavidin was added to the wells and incubated for 1hr at room temperature. Next the microplates were washed, TMB (Bethyl laboratories Inc.) was added to the wells. When peroxidase activity was revealed, 1M H2SO4 was added to the wells to stop the reaction and the optical density was measured (at 450nm) using a spectrophotometer (Tecan VVI 13512R spark) and the data was analyzed by Deltasoft JV. The IL-1β, IL-12, IL-6, TNF-α, IL-10 ELISA’s were ordered from R&D systems (respectively DY681, DY912, DY686, DY690B, DY693B) and carried out following the manufacturer’s instructions. The optical density was measured (at 450nm) using a spectrophotometer (Tecan VVI 13512R spark) and the data was analyzed by Deltasoft JV.

### 2.5 Flow cytometry

PBMC were stained for CD4 and CD172a cell surface expression. The pDC population was determined based on their forward scatter (FSC), side scatter (SSC) and CD4^+^ CD172a^dim^ phenotype. The general lymphocyte compartment was selected based on FSC, SSC and the absence of CD172a expression, and monocytes were identified based on FSC, SSC and their CD172a^high^ phenotype. Anti-CD4 (clone 74-12-4) and anti-CD172a (clone 74-33-15) monoclonal antibodies were a kind from Dr. A. Saalmüller (University of Vienna, Austria) and were described before (31). The PBMC population was incubated with the primary antibodies for 20 minutes at 4°C (biotinylated mouse anti-porcine CD4, mouse anti-porcine CD172a). Cells were washed three times with PBS with 1% EDTA (Life Technologies). Then, cells were incubated for 20 min with the secondary antibodies (streptavidin R-PE, Invitrogen ref:SA10041; anti-mouse IgG1 Alexa fluor 647, Invitrogen ref:A21240). Cells were again washed three times and incubated with propidium iodide (Invitrogen ref:P3566). Cells were analyzed using a Novocyte flow cytometer (Agilent technologies).

### 2.6 Statistical analysis

All experiments were subjected to statistical analysis as indicated in the figure legends, statistical analysis was performed using GraphPad Prism 9.0 software.

## 3 Results

### 3.1 Age-dependent differences in IFN-α production by PBMC/pDC in response to PRV-infected cells or a TLR9 agonist

**Figure 1:**
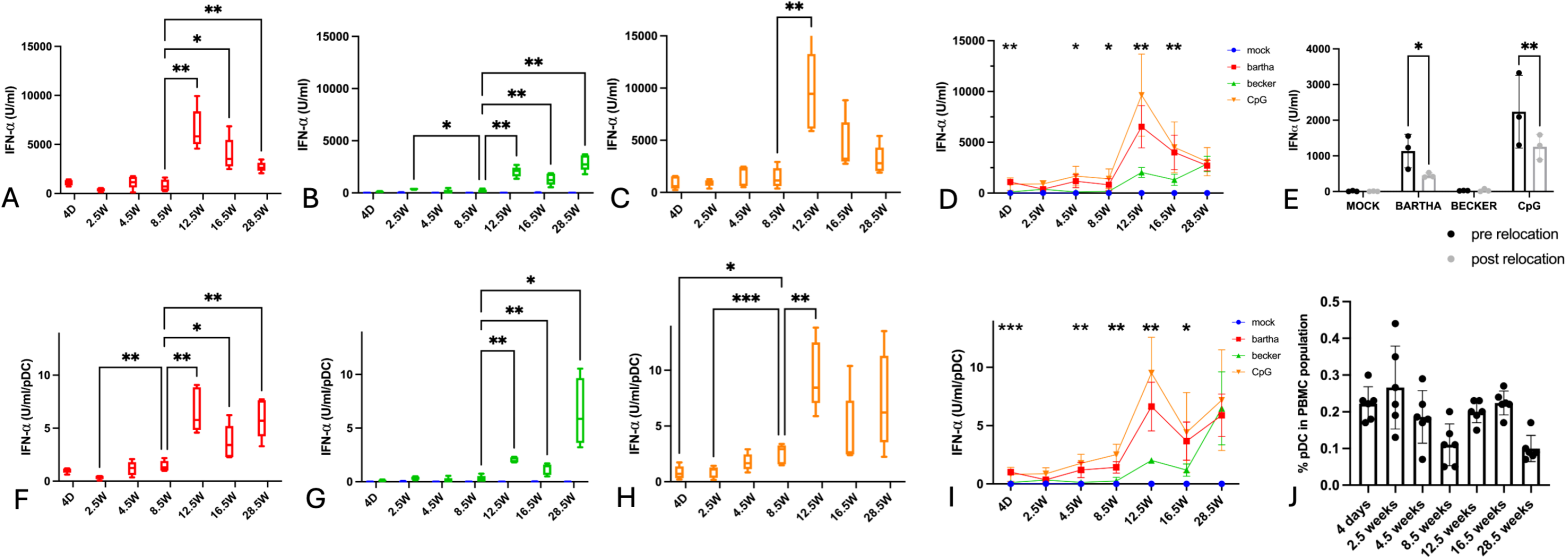
Total IFN-α production by PBMC and IFN-α production per pDC in response to PRV Bartha, PRV Becker or the TLR9 agonist CpG increases substantially after the age of 8,5 weeks. ST cells, infected with PRV Bartha (red) **(A, D)**, Becker (green) **(B, D)** or mock infected (blue) **(A–D)** were incubated with PBMC from piglets at different ages. An additional condition where PBMC were added to ST cells together with the TLR9 agonist CpG ODN32 was also included (orange) **(C, D)**. After 22hrs of incubation, supernatant was collected and IFN-α levels were determined by ELISA. Graphs **(A-D)** show total IFN-α production. **(E)** shows the IFN-α response by porcine PBMC towards the different stimuli before and after relocation at 12.5 weeks of age. Percentage of pDC in each PBMC sample in **(D)** was determined using flow cytometry **(J)**) and was used to calculate the IFN-α produced per pDC figure **(F-I)**. Asterisks in candlestick charts represent statistically significant differences of individual conditions compared to the 8.5 weeks of age condition. Asterisks in figure **(D, I)** represent significant differences between Bartha and Becker conditions at different timepoints. Graphs show means, standard deviations and individual data points of 5 independent repeats (3 repeats in **(E)**). (*, P < 0.05, **, P< 0.01, ***, P<0.001, using two way ANOVA).

Blood samples were collected from 5 piglets, starting at 4 days of age. Samples were collected every two weeks until 4.5 weeks of age, subsequently at four weeks intervals up to 16.5 weeks of age and then a final time at 28.5 weeks of age. From each sample, PBMC were isolated using a lymphoprep density gradient and incubated for 22h with swine testicle (ST) cells that were either mock-infected or infected with the virulent wild type PRV strain Becker or the attenuated PRV vaccine strain Bartha. Since the IFN-α response by PBMC/pDC against alphaherpesviruses like HSV and PRV predominantly occurs via TLR9 (20, 32), we also included a condition where PBMC were stimulated with ST cells treated with the TLR9 agonist CpG ODN32. After incubation for 22h, the amount of IFN-α produced in the supernatant was quantified, which provided the total IFN-α production by the PBMC (**Figures 1A–D**). Flow cytometric analysis was also performed on the PBMC populations of each sample to determine the percentage of plasmacytoid dendritic cells (pDCs). Although the percentage of pDCs in PBMC fluctuated somewhat with age, no clear age-dependent differences in the relative size of the pDC population within PBMC was observed (**Figure 1J**).

Since the same amount of PBMC was used in every condition (500,000 PBMC/well) and since the relative abundance of pDC within each PBMC sample was determined by flow cytometry (**Figure 1J**), it was possible to calculate the number of pDCs included in each assay. Previous studies have demonstrated that virtually all IFN-α produced by PBMC in response to PRV-infected ST cells originates from pDCs (15). By taking both the total IFN-α production by PBMC and the percentage of pDCs per PBMC sample into account, an estimate of the amount of IFN-α produced per pDC could be calculated for each sample (**Figures 1E–H**).

Our findings reveal clear age-dependent changes in total IFN-α production by PBMC and the calculated amount of IFN-α produced per pDC in response to PRV-infected ST cells (**Figure 1**).

During the first 8.5 weeks of life, the total IFN-α production in response to either PRV strain or CpG remained low. Strikingly, during that period, the IFN-α production in response to wild-type PRV strain Becker-infected ST cells was virtually undetectable and not significantly different from that against mock-infected ST cells (**Figures 1B, D**). After 8.5 weeks of age, the IFN-α response increased substantially, with a distinct peak at 12.5 weeks of age. At all time points, the IFN-α response against wild type PRV-infected cells was substantially lower than that against attenuated Bartha-infected cells, except in the adult pigs (28,5 weeks, **Figure 1D**).

A similar evolution was also evident in the calculated amount of IFN-α produced per individual pDC, which also rose significantly after 8.5 weeks of age (**Figures 1E–H**).

Total IFN-α production by PBMC in response to Bartha-infected or CpG-treated cells declined from 12.5 up to 28.5 weeks of age (whereas it remained relatively stable at low levels in response to Becker-infected cells) (**Figures 1A, C, D**). Interestingly, the calculated production capacity per pDC against Bartha-infected or CpG-treated cells remained relatively stable between 16.5 and 28.5 weeks of age, and was even increased at 28.5 weeks of age in response to Becker-infected cells (**Figures 1E, G, H**).

These data reveal a robust age-dependent increase in IFN-α production by porcine PBMC/pDC in response to PRV-infected or CpG-treated cells, with a clear breaking point between 8.5 and 12.5 weeks of age. In addition, the IFN-α response by PBMC/pDC against wild type PRV-infected cells was virtually undetectable in pigs up to the age of 8.5 weeks.

At 12.5 weeks of age, the piglets were transferred to the Faculty of Veterinary Medicine. To ensure that this relocation did not affect interferon responses, a control experiment was performed. Blood samples were collected from three piglets both before and after relocation. PBMC IFN-α responses were compared, revealing a mild suppressive effect on the interferon response following transport (**Figure 1E**). Hence, the increased IFN-α response at 12.5 weeks of age in the longitudinal assay can not be attributed to relocation effects.

### 3.2 Age-dependent evolution in IL-12 production by PBMC in response to PRV-infected cells or a TLR9 agonist

**Figure 2:**
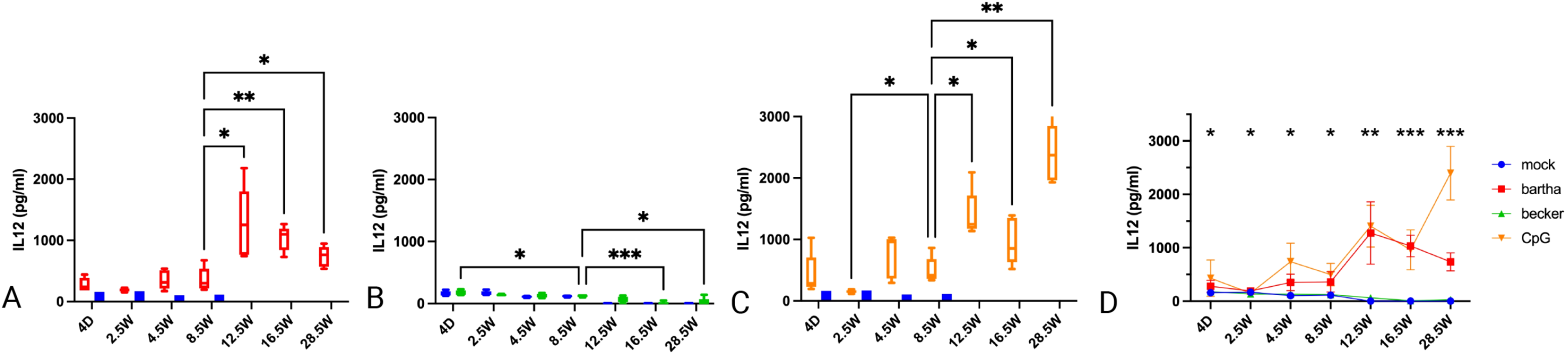
IL12 production by PBMC in response to PRV Bartha, Becker or the TLR9 agonist CpG, depending on the age of the piglets. ST cells, infected with PRV Bartha (red) **(A, D)**, Becker (green) **(B, D)** or mock infected (blue) **(A–D)** were incubated with PBMC from piglets at different ages. An additional condition where PBMC were added to ST cells together with the TLR9 agonist CpG ODN32 was also included (orange) **(C, D)**. After 22hrs of incubation, supernatant was collected and IL12 levels were determined by ELISA. After 8.5 weeks of age, IL12 production increased substantially in response to the Bartha strain or CpG, but not to the wild type PRV strain Becker. Graphs show means, standard deviations and individual data points of 5 independent repeats. Asterisks in candlestick charts represent statistically significant differences of individual conditions compared to the 8.5 weeks of age condition. Asterisks in **(D)** represent significant differences between Bartha and Becker at different time points. (*, P < 0.05, **, P< 0.01, ***, P<0.001, using two way ANOVA).

In all conditions, IL-12 production remained relatively low up to 8.5 weeks of age. From 12.5 weeks onwards, IL-12 production by PBMC substantially increased in response to Bartha-infected or CpG-treated cells (**Figures 2A, C, D**). Remarkably, the IL-12 response against Becker-infected cells was very low to undetectable (and not different from that against mock-infected cells) at all time points (**Figure 2B**). Hence, the IL-12 response by PBMC against Bartha-infected or CpG-treated cells in general follows a similar pattern as that observed for IFN-α. However, whereas the IFN-α response against Becker-infected cells was virtually undetectable up to the age of 8.5 and then increased, IL-12 responses against Becker-infected cells remained virtually undetectable up to the age of 28.5 weeks of age, the last tested time point (**Figure 2B**).

### 3.3 Age-dependent evolution in IL-1-β production by PBMC in response to PRV-infected cells or a TLR9 agonist

**Figure 3:**
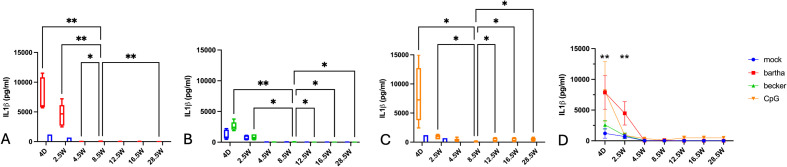
IL-1-β production by PBMC in response to PRV Bartha, Becker or the TLR9 agonist CpG, depending on the age of the piglets. ST cells, infected with PRV Bartha (red) **(A, D)**, Becker (green) **(B, D)** or mock infected (blue **(A–D)** were incubated with PBMC from piglets at different ages. An additional condition where PBMC were added to ST cells together with the TLR9 agonist CpG ODN32 was also included (orange) **(C, D)**. After 22hrs of incubation, supernatant was collected and IL-1-β levels were determined by ELISA. Graphs show means, standard deviations and individual data points of 5 independent repeats. Asterisks in candlestick charts represent statistically significant differences of individual conditions compared to the 8.5 weeks of age condition. Asterisks in **(D)** represent significant differences between Bartha and Becker at different time points. (*, P < 0.05, **, P< 0.01, using two way ANOVA).

In contrast to the production of IFN-α and IL-12, production of IL-1-β by PBMC in response to PRV-infected or CpG-treated ST cells was highest in newborn piglets (4 days of age) (**Figures 3A–D**). This strong response at 4 days of age was most pronounced against the attenuated Bartha vaccine virus and the TLR9 agonist CpG but was also evident in response to the virulent PRV Becker strain. The IL-1-β response decreased at 2.5 weeks of age and was virtually undetectable from 4.5 weeks of age onwards. Even in the mock condition, a slight IL-1-β production could be noticed in newborn piglets (**Figure 3**). Hence, PBMC of newborn piglets have a propensity to produce large amounts of IL-1-β, in particular in response to Bartha-infected or CpG-treated cells, whereas PBMC derived from pigs from ages 4.5 weeks and onwards produced negligible amounts of IL-1-β in all of the tested conditions.

### 3.4 Age-dependent evolution in IL-6 production by PBMC in response to PRV-infected cells or a TLR9 agonist

**Figure 4:**
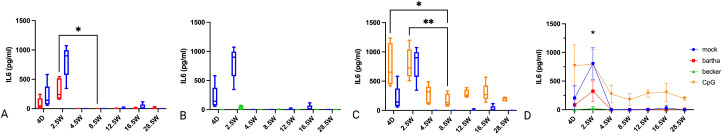
IL-6 production by PBMC in response to PRV Bartha, Becker or the TLR9 agonist CpG, depending on the age of the piglets. ST cells, infected with PRV Bartha (red) **(A, D)**, Becker (green) **(B, D)** or mock infected (blue) **(A–D)** were incubated with PBMC from piglets at different ages. An additional condition where PBMC were added to ST cells together with the TLR9 agonist CpG ODN32 was also included (orange) **(C, D)**. After 22h of incubation, supernatant was collected and IL-6 levels were determined by ELISA. Graphs show means, standard deviations and individual data points of 5 independent repeats. Asterisks in candlestick charts represent statistically significant differences of individual conditions compared to the 8.5 weeks of age condition. Asterisks in **(D)** represent significant differences between Bartha and Becker at different time points. (*, P < 0.05, **, P< 0.01, using two way ANOVA).

IL-6 production was highest in PBMC from very young piglets, at 4 days and 2.5 weeks of age (**Figure 4**). This response was even observed against mock-treated cells and was highest in response to CpG-treated cells. The IL-6 response to Bartha-infected cells was similar to that against mock-treated cells, whereas the IL-6 response against wild type Becker PRV-infected cells was virtually absent at all time points, and thus even lower than that against mock-treated cells. From 4.5 weeks of age onwards, a detectable IL-6 response was only observed against CpG-treated cells (**Figure 4**). Hence, PBMC of young piglets are prone to high production of IL-6. However, PRV-infected cells do not appear to trigger an increased IL-6 response by PBMC over that observed against mock-treated cells and, when using virulent PRV strain Becker, even display lower IL-6 responses than observed in the mock condition.

### 3.5 Age-dependent evolution in TNF-α production by PBMC in response to PRV-infected cells or a TLR9 agonist

**Figure 5:**
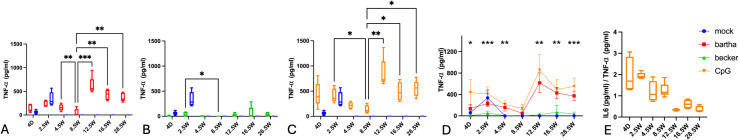
TNF-α production by PBMC in response to PRV Bartha, Becker or the TLR9 agonist CpG, depending on the age of the piglets. ST cells, infected with PRV Bartha (red) **(A, D)**, Becker (green) **(B, D)** or mock infected (blue) **(A–D)** were incubated with PBMC from piglets at different ages. An additional condition where PBMC were added to ST cells together with the TLR9 agonist CpG ODN32 was also included (orange) **(C, D)**. After 22h of incubation, supernatant was collected and TNF-α levels were determined by ELISA. Graphs show means, standard deviations and individual data points of 5 independent repeats. Asterisks in candlestick charts represent statistically significant differences of individual conditions compared to the 8.5 weeks of age condition. Asterisks in **(D)** represent significant differences between Bartha and Becker at different time points. **(E)** represents the IL6/TNF-α ratio for PBMC stimulated with CpG at different ages. (*, P < 0.05, **, P< 0.01, ***, P<0.001, using two way ANOVA).

Like for IL-6, TNF-α production by PBMC of young piglets (4 days and 2.5 weeks of age) is fairly high, even in response to mock-treated cells (**Figure 5**). Like for IL-6, the TNF-α response by PBMC of these young animals against Bartha-infected cells is comparable to that against mock-treated cells (**Figure 5A**), while the TNF-α response against CpG is higher and the response against virulent Becker-infected cells is virtually undetectable (and lower than that against mock-treated cells) (**Figures 5B, C**). From 4.5 weeks of age, the TNF-α response against mock-treated cells is undetectable and is also decreased (but still noticeable) in response to Bartha-infected or CpG-treated cells. Of note, like the IFN-α and IL-12 response, the TNF-α response against Bartha-infected cells and CpG-treated cells shows a substantial increase at 12.5 weeks of age and then gradually wanes off. Remarkably, again, the TNF-α response of PBMC in response to wild type Becker PRV-infected cells remains very weak to undetectable at all ages (**Figures 5B, D**). The IL6/TNF-α production ratio for PBMC stimulated with CpG consistently decreases with increasing age (**Figure 5E**).

### 3.6 Age-dependent evolution in IL-10 production by PBMC in response to PRV-infected cells or a TLR9 agonist

**Figure 6:**
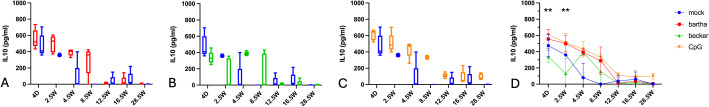
IL-10 production by PBMC in response to PRV Bartha, Becker or the TLR9 agonist CpG, depending on the age of the piglets. ST cells, infected with PRV Bartha (red) **(A, D)**, Becker (green) **(B, D)** or mock infected (blue) **(A–D)** were incubated with PBMC from piglets at different ages. An additional condition where PBMC were added to ST cells together with the TLR9 agonist CpG ODN32 was also included (orange) **(C, D)**. After 22h of incubation, supernatant was collected and IL-10 levels were determined by ELISA. Graphs show means, standard deviations and individual data points of 5 independent repeats. Asterisks in candlestick charts represent statistically significant differences of individual conditions compared to the 8.5 weeks of age condition. Asterisks in **(D)** represent significant differences between Bartha and Becker at different time points. (**, P< 0.01, using two way ANOVA).

Across all conditions, IL-10 production is high very early in life (**Figure 6**). Notably, even under mock conditions, a significant amount of IL-10 is produced. However, IL-10 levels in the mock condition begin to decline from 2.5 weeks onwards. In response to both CpG and Bartha stimulation, IL-10 production markedly decreases after 8.5 weeks of age. From 12,5 weeks onwards, the IL-10 production is fairly low in all conditions, and virtually undetectable in response to Bartha- or Becker-infected cells.

## 4 Discussion

In the current study, we report very substantial age-dependent differences in the cytokine response profile of primary porcine PBMC to cells infected with PRV that correlate with well-known changes in symptomatology of PRV in pigs of different ages.

The production of IFN-α and IL-12 against PRV-infected cells and the TLR9 agonist CpG remains low up until the age of 8.5 weeks, especially for the virulent strain Becker (**Figure 1**). These findings align with the observed shift in symptomatology. In piglets under 9 weeks of age, disease is characterized by neurological symptoms and a high mortality especially in piglets under 3 weeks of age, while pigs older than 9 weeks exhibit milder symptoms, such as respiratory issues including sneezing, nasal discharge, and coughing (6–9). Different studies have shown that robust IFN-α production early in infection is required for suppressing viral replication and cytopathic effect in herpesvirus infections (11–14).

A timely and efficient type I IFN response appears to be critical to prevent aggravated and potential life-threatening disease for different virus infections, including SARS-CoV-2. A delayed or insufficient IFN-α response has been associated with higher viral replication, excessive release of pro-inflammatory cytokines like IL-6 and TNF-α, and the onset of a cytokine storm. This dysregulated immune activation contributes to systemic inflammation and severe pathology, including fatal consequences (33, 34). Studies have shown that elevated serum levels of inflammatory cytokines such as IL-6 and TNF-α at the time of hospitalization are strong negative prognostic markers in COVID-19 patients (35). These findings are consistent with observations in other viral infections, such as generalized herpesvirus infections in neonates, where higher levels of IL-6 and TNF-α correlate with poorer outcomes (36, 37). This suggests that immunopathological damage, rather than the direct effects of the virus, are often the primary contributors to severe disease manifestations (37). In pigs, the detrimental effects of such a cytokine storm has also been documented in domestic pigs infected with virulent African swine fever virus, where delayed innate immune responses were implicated in the onset of severe systemic inflammation (38).

Historically, the increased susceptibility of newborns to viral infection was ascribed to ‘immaturity’ of the immune cells. With continuous research of the neonatal immune system, it was found that the reality is more nuanced and differences in cytokine profiles of newborns versus adults are determining factors in pathogen susceptibility (39). In humans, newborns produce a much higher IL-6/TNF-α ratio in response to TLR triggers than adults (39). Our results show that this ratio follows a similar trend in piglets in response to the TLR9 agonist CpG ODN32 (**Figure 5E**). In humans, a basal amount of IL-6 production was even measured in uninfected newborns, possibly induced by exposure to environmental TLRs and colonizing bacteria (39). IL-6 has Th2/Th17 polarizing properties, whereas TNF-α steers the immune response towards a Th1 polarized immune response. A higher IL-6/TNF-α ratio in newborns will thus favor Th2 dominated immune responses and reduced Th1 functionality (39–44). In addition to this, human neonates and young children also show a significantly reduced capability to produce adequate amounts of IL-12 (45–47). IL-12 is another important cytokine in steering the immune system towards a Th1 biased response (45–50). In line with this, herpes simplex infection in neonates is known to trigger vast amounts of IL-6 and inadequate amounts of IL-12 and IFN-α, resulting in a Th2 dominated immune response (50, 51). This is unfavorable, as Th1 responses are crucial in the defense against intracellular pathogens such as herpesviruses (27, 41, 52, 53), and may help to explain why newborns are more susceptible to severe herpesvirus infections and infections with intracellular pathogens in general. Our current findings support these hypotheses and notions, as we observed higher production of IL-6 in newborn piglets. Even unstimulated PBMCs produced substantial amounts of IL6, as was reported in humans (39). Production of IL-6 decreases with age of the piglets. As in humans, IL-12 production is severely reduced in the newborn piglets, and remains low up until 8.5 weeks of age. Despite the relatively high TNF-α levels, the high IL-6 and low IL-12 cytokine profile in newborn piglets may also suggest a Th2/Th17 dominant environment and reduced Th1 stimulation (50, 54, 55). Assessing the production of IL-4 and IL-17 cytokines to further support this hypothesis would be valuable in future research.

In line with our other results, IL-10 production was significantly higher in the younger individuals compared to the older animals. This higher production of IL-10 in younger subjects was also seen in human and mice (56–59). IL-10 is known to be an anti-inflammatory and Th2 skewing cytokine, further supporting our current hypothesis (60, 61). In humans, IL-10 is known to suppress cells of the myeloid lineage by transcriptional inhibition of pro-inflammatory cytokines such as interferons and IL-12 (61)Interestingly, our data show that IL-10 levels start decreasing significantly after 8.5 weeks of age and this coincides with the observed increased levels of IFN-α, TNF- α and IL-12. Moreover, increased serum levels of IL-10 were also correlated with age-dependent impaired immune responses to infections in mice (62).

The most notable difference in cytokine response of porcine PBMC against PRV or CpG was observed between 8.5 and 12.5 weeks of age. Physiologically, notable immunological changes occur in piglets around 8 weeks of age. Through colostrum—and to a lesser extent, during the lactation period—piglets receive maternal antibodies and immune cells from the sow (63). Due to their natural half-life, these maternal immune components gradually decline over the first weeks of life. For most viruses and pathogens, maternal immunity reaches its lowest point between 6 and 8 weeks of age (63). During this window, the piglet’s own immune system becomes increasingly exposed to environmental pathogens, thereby triggering endogenous immune activation. This increased antigenic stimulation may possibly contribute to the observed peak in cytokines such as IFN-α, TNF-α, and IL-12 in the period shortly thereafter. Although speculative, it will be interesting to assess whether “trained immunity” of myeloid cells could be involved. Trained immunity is the phenomenon where endogenous or exogenous stimuli evoke reprogramming of the innate immune cells through epigenetic changes in pro-inflammatory regions (64, 65). Although such phenomenon could represent a physiological shift in immune regulation and may serve as a working hypothesis to explain higher TNF-α, IFN-α and IL12 responses and the onset of a more Th1-skewed immune response at this age, at this point, this hypothesis remains purely speculative.

IL-1β levels are high in young animals, even under mock conditions, and tend to decline with age. A similar trend has been observed in humans, where elevated IL-1β levels shortly after birth are thought to result from the physiological stress of birth and the abrupt environmental changes that follow (66). In piglets, it has also been demonstrated that significant amounts of IL-1β are transferred from the sow to the offspring via colostrum (67, 68), which may explain the substantial IL-1β levels observed even in mock-treated samples. The reason why IL-1β production by PBMCs in response to viral stimulation markedly decreases during the first few weeks of life remains unclear at this point. IL-1β is primarily produced by monocytes (69) and in humans, neonates have a higher proportion of circulating monocytes compared to adults (53). In line with this, we observed that, except for the 2.5 weeks time point, the relative percentage of monocytes in PBMC was high in young piglets up to 8.5 weeks of age, followed by a general decreasing trend (**Supplementary Figure S1**). Although speculative, this higher percentage of circulating monocytes in young piglets could potentially contribute to the higher IL-1β production in neonates, although further research is needed to investigate this.

It will also be interesting in future research to more generally assess the contribution of individual immune cell populations, such as pDC and monocytes, to the observed age-dependent differences in cytokine production. For example, it may be interesting to investigate to what extent the age-related decline in the percentage of monocytes (**Supplementary Figure S1**), may possibly contribute to the observed decreases in IL-6 and IL-10 production, as these cytokines can also be produced by monocytes (70, 71). Regarding the general lymphocyte compartment, except for a moderate, temporal drop in its percentage around 4.5 weeks of age, this compartment appeared to be quite stable (**Supplementary Figure S1**), which may suggest that differences in general lymphocyte percentages may not be a major factor driving the observed age-dependent differences in cytokine responses.

As an important side-note, the current IL12 data need to be interpreted with some caution, as the ELISA was performed on the IL-12 p40 subunit. IL-12 is a heterodimer that shares its p40 subunit with IL-23, making it challenging to distinguish between IL-12 and IL-23 when performing an ELISA detecting p40 (72, 73). PBMCs have the ability to produce IL-23, although the IL-23 secretion by adult PBMC is suppressed by TLR-9-induced IFN-α (74), suggesting that the IL12p40 levels that are detected in PBMC from older pigs (with strong IFN-α responses) likely can be mainly attributed to IL-12. Moreover, studies in human neonates have shown that IL-23 production is higher at birth and subsequently decreases with age (53). Since our studies indicate an increase in IL12p40 production with increasing age, this further suggests that the IL12p40 signal is mainly derived from IL12 rather than IL23. Another limitation of the current study is the fact that the cytokine profiles were investigated in an artificial *in-vitro* set-up. We have worked with infected swine epithelial cells to closely mimic the *in-vivo* situation. Still, we cannot fully mimic the complexity of interactions between different immune cells, tissues and signaling molecules. Thus, it will be valuable to further investigate to which extent the current findings are reproducible to the actual *in-vivo* situation.

Herpesviruses are known to suppress the host immune response in a myriad of ways (75–77). We have reported earlier that virulent, wild type PRV suppresses IFN-α production by plasmacytoid dendritic cells (pDC) (15, 20, 75, 76) and that the ability to suppress IFN-α production by pDC is substantially impaired in the most widely used attenuated PRV vaccine strain Bartha (15). Interestingly, we now show that the ability of the WT PRV strain Becker to suppress IFN-α production may be age dependent. In younger individuals up until 16.5 weeks of age, Bartha elicits a significantly higher IFN-α response by PBMC compared to the wild type Becker strain. In adult pigs (28.5 weeks), the IFN-α response by PBMC to Becker increases to a level that is similar to that observed for Bartha. In line with this, the amount of IFN-α produced per pDC in response to Bartha is higher than the amount produced in response to Becker in young piglets, but comparable in adults. These data indicate that the pDC-suppressing properties of PRV may be less effective in adult pigs, which display reduced PRV disease severity (6–9).

In this study, animals were sourced from a single litter, which helped reduce variability due to environmental and early-life differences. While this design improves internal consistency, this setup may reduce the independence of observations, as animals share both genetic background and early maternal influences. At 12.5 weeks of age, the piglets were transferred to the Faculty of Veterinary Medicine. To verify whether transport had any effect, in a separate group of piglets of 12 weeks old, IFNα responses were compared before (control) and immediate after transport. Blood samples were collected immediately prior to and directly after transportation (the latter is similar to what was done at the 12.5 week time point in the longitudinal assay). We observed that transport exerted a slight suppressive effect on the IFNα response (**Figure 1E**). Given that an increase in IFN-α production was observed at 12.5 weeks of age in the longitudinal study, it can be concluded that this rise cannot be attributed to a relocation effect.

In humans, age-dependent differences have been reported in additional innate immune factors, that could possibly be involved in different susceptibility to (herpes)virus infections. For example, human neonates exhibit a higher proportion of circulating monocytes compared to adults (53). In contrast, adults possess a higher percentage of conventional dendritic cells (cDCs) (53), contributing to a more efficient antigen presentation capacity. Neonates also display an underdeveloped complement system, reduced leukocyte extravasation, and impaired chemotactic responses (78), which all may contribute to decreased pathogen clearance. Of interest, while neonatal natural killer (NK) cells are more abundant in younger individuals, they exhibit markedly reduced cytotoxic activity and responsiveness to HSV-infected cells (79–82). Likewise, NK cells are also more abundant in piglets compared to older pigs (26) and piglets NK cells are hyporesponsive to interferon stimulation, leading to reduced cytotoxicity towards infected cells (83). Hence, in future research, it will be interesting to assess potential age-dependent responses against PRV for other innate immune effectors, particularly NK cell-mediated cytotoxicity and IFN-γ production.

At present, it is unclear why the production level of some cytokines is markedly lower in PBMC from young pigs compared to those of older pigs. Since we observed that IFN-α responses to both viral stimuli and CpG exhibit similar trends, we hypothesize that developmental changes within the TLR9 signaling cascade may contribute to this difference. Future research will clarify whether, for example, differences in expression levels and/or activity of TLR9 and/or downstream transcription factors such as IRF7 may be involved. Interestingly, our results are to some extent in line with those of Vreman and colleagues, who also showed lower IFN-alpha and TNF-alpha protein levels in the supernatant of CpG-stimulated PBMC from neonatal versus older pigs (84). The authors did not observe age-dependent differences in mRNA levels of IFN-alpha or TNF-alpha in white blood cell populations enriched for pDC (84). In human pDCs, it has been shown that although IRF7 expression levels are comparable between neonates and adults, the ability of IRF7 to translocate to the nucleus and initiate interferon transcription is significantly impaired in neonates (85). Another possible speculative explanation for the distinct cytokine profiles observed in young versus adult pigs involves epigenetic regulation. In humans, for instance, the Th2 locus in CD4^+^ T cells is known to be hypomethylated during early life, resulting in increased expression of Th2 cytokines. Conversely, loci associated with Th1 responses, such as the IFN-γ gene, are often hypermethylated, thereby reducing their expression (79, 86). Whether similar epigenetic modifications occur in pigs remains unknown and will be the focus of future assays. Of note, the current data were obtained in PBMC. Future assays on purified pDC will clarify to what extent our data indicate age-dependent differences in pDC functionality and/or effects on other immune cells that may possibly indirectly affect pDC functionality.

The current research helps to gain a better understanding of the immune system of newborns, which in turn may aid in determining optimal timing for vaccination programs for viral infections, as inferior vaccine responses are a returning issue when vaccinating young individuals (48). Currently, pigs are vaccinated against PRV at 10 weeks of age (87). Our data are in support of this timing, as they indicate that pigs of 8.5 weeks of age or younger may elicit low levels of IFN-α and IL12 upon vaccination, while these cytokines are important drivers to elicit effective Th1 responses and lasting cellular immunity. In addition, our data may also provide insights in and provide a model system for potential therapeutic applications. Indeed, therapeutically modulating the newborn’s immune system towards a Th1 biased immune response either in human or pig, by administering specific cytokines or adjuvants, could ameliorate the neonatal antiviral defense mechanisms. This could also have implications later in life, as it has been demonstrated that neonates who experience severe infections early in life and respond with a Th2-dominated response are more likely to have a Th2-biased immune profile later in life, making them more susceptible to intracellular pathogens (46). Hence, it will be interesting in future research to assess whether particular cytokines/additives may affect the cytokine profile of PBMC of young piglets towards PRV/CpG and thereby may show promise as early intervention drugs to improve the neonatal immune response against life-threatening viral infections.

## Data Availability

The original contributions presented in the study are included in the article/**Supplementary Material**. Further inquiries can be directed to the corresponding author.
