# Supplementary figures and images for: Age-dependent differences in type I interferon, IL-12 and pro-inflammatory cytokine production by porcine peripheral blood mononuclear cells in response to pseudorabies virus-infected cells
Source: Front Immunol. 2025 Jul 3;16:1596490. doi: 10.3389/fimmu.2025.1596490 (PMC12267268; doi:10.3389/fimmu.2025.1596490)

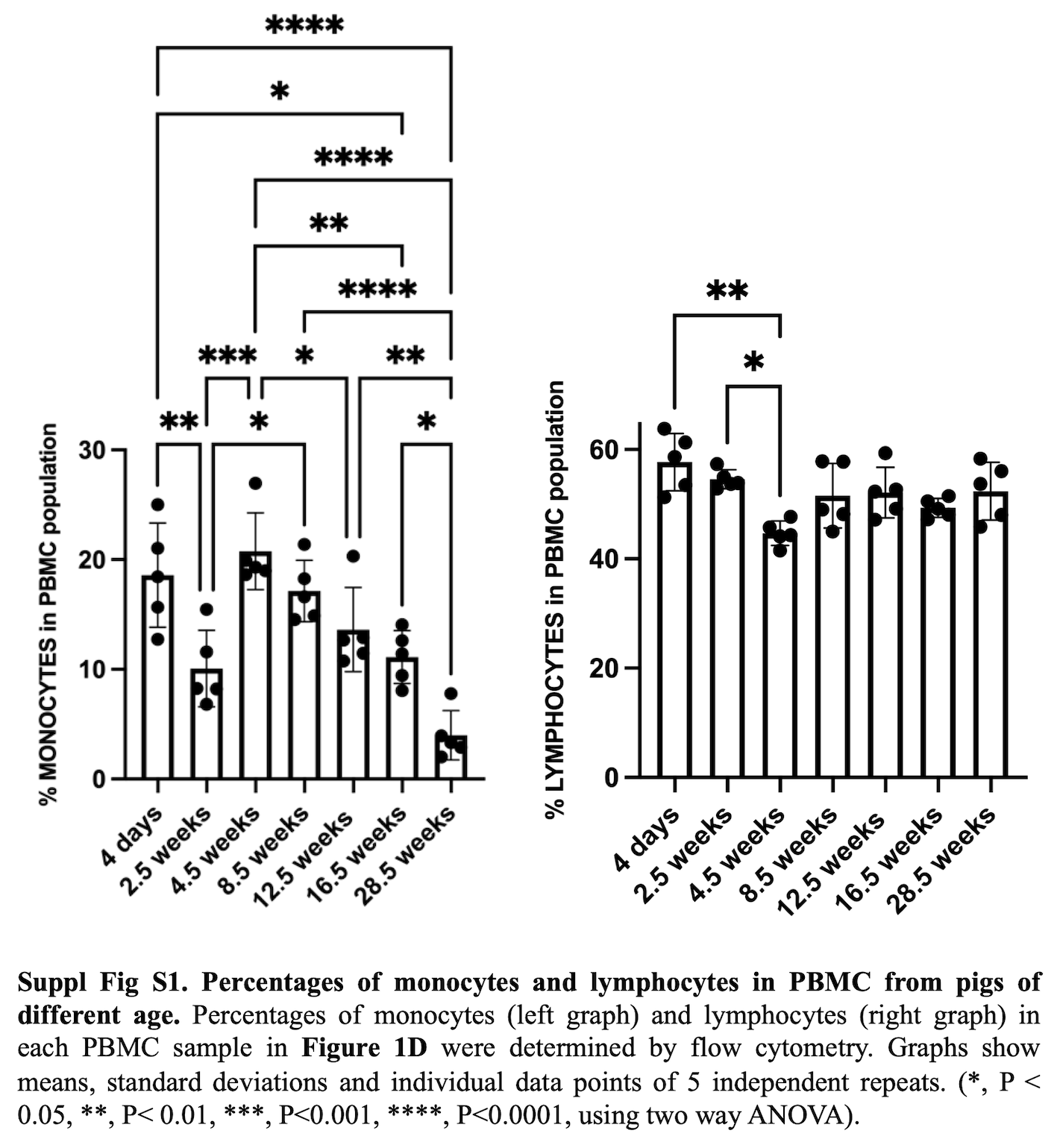

Supplement: Supplementary file 1 [file Image1.tiff]
